# RADFRNet: Detail-Enhanced Feature Recalibration for Infrared Small-Target Detection Based on an Improved YOLOv11

**DOI:** 10.3390/s26154850

**Published:** 2026-08-01

**Authors:** Chenyang Li, Jie Cao, Qun Hao, Chenghao Song, Haifeng Yao, Zhipeng Wei

**Affiliations:** 1 School of Opto-Electronic Engineering, Changchun University of Science and Technology, Changchun 130022, China; 2025200391@mails.cust.edu.cn (C.L.); 2024004858@mails.cust.edu.cn (C.S.); scifeng@cust.edu.cn (H.Y.); weizp@cust.edu.cn (Z.W.); 2 School of Optics and Photonics, Beijing Institute of Technology, Beijing 100081, China; 3 Yangtze Delta Region Academy, Beijing Institute of Technology, Jiaxing 314003, China

**Keywords:** infrared small-target detection, multi-class infrared detection, YOLOv11-n, differential convolution, cross-level feature recalibration

## Abstract

Infrared multi-class small-target detection is challenging because targets occupy few pixels, exhibit weak texture, and are easily confused with background clutter. We present RADFRNet, a YOLOv11-n-based detector designed to address two forms of information degradation: detail loss in the backbone and semantic–spatial mismatch during cross-level feature fusion. First, the previously proposed DEConv operator is embedded into selected C3K2 stages to form C3DEConv; the contribution lies in its C3K2-compatible, detector-oriented integration rather than in a new differential-convolution formulation. Second, an Adaptive Feature Recalibration (AFRE) block constructed from three Recalibration Attention Units performs bidirectional interaction between shallow spatial details and deep semantic features. We also construct four-class bounding-box annotations for BIT-SIRST. RADFRNet achieves mAP@0.5 scores of 93.2% and 65.3% on BIT-SIRST and FLIR-ADAS-v2, improving YOLOv11-n by 4.4 and 8.5 percentage points, respectively. Under the same original 640 × 640 GPU inference setup, the per-image latency increases from 3.3 to 6.7 ms on BIT-SIRST and from 3.0 to 7.8 ms on FLIR-ADAS-v2, corresponding to nominal throughputs of approximately 149 and 128 FPS for RADFRNet. The reported model-complexity values are 8.2 M and 8.6 M, respectively. These results show that RADFRNet retains high-rate GPU inference, although the accuracy gains are obtained at a clear computational cost; the model is therefore positioned as an accuracy-oriented detector rather than a latency-neutral replacement for YOLOv11-n.

## 1. Introduction

Infrared multi-class small-target detection uses radiation differences between targets and their surroundings to locate objects of interest. The technique is important in military and civilian applications, including infrared search-and-track, battlefield assessment, traffic monitoring, agricultural monitoring, and pest surveillance [1,2,3,4,5,6]. Developing detectors that balance accuracy and latency is therefore practically important.

An infrared small target typically appears with low contrast, occupies only a few pixels, and provides limited texture and shape cues [7,8,9]. Its features are easily overwhelmed by clutter and noise, leading to missed detections and false alarms. These difficulties become more pronounced in dense scenes and after repeated downsampling in deep detection networks. Large-receptive-field operators, including wavelet-based convolutions, provide an additional route for retaining multiscale context [10].

YOLOv11-n adopts an anchor-free detection framework with C3K2-based feature aggregation and a decoupled detection head, providing a favorable speed–accuracy trade-off. However, two limitations remain in infrared small-target detection. First, repeated downsampling can weaken the already limited boundary and gradient cues of tiny targets. Second, direct concatenation in the FPN–PAN neck does not explicitly resolve the mismatch between noisy shallow features and semantically strong but spatially coarse deep features. To address these two failure modes, we construct RADFRNet. The previously proposed DEConv operator is embedded into selected C3K2 stages to form C3DEConv, while a three-RAU Adaptive Feature Recalibration (AFRE) block replaces direct fusion at the cross-level nodes of the neck. The two components target different stages of information degradation: C3DEConv preserves edge-sensitive features in the backbone, and AFRE recalibrates shallow and deep representations during feature fusion.

The main contributions are as follows:

(1) We construct four-class bounding-box annotations for BIT-SIRST, covering car, person, UAV, and ship targets. The revised dataset description reports only information that can be verified from the retained annotation set and does not claim a numerical inter-annotator agreement rate that cannot be reproduced from the available records.
(2) We develop a C3K2-compatible detail-enhanced aggregation module, termed C3DEConv, by integrating the previously proposed DEConv operator into selected stages of the YOLOv11-n backbone. The contribution is not a new differential-convolution formulation; it lies in structural integration with C3K2, stage-specific deployment, and adaptation to weak-texture infrared targets. The training-time branches are merged into an equivalent convolution for deployment, avoiding branch-wise execution.
(3) We construct an AFRE block using three Recalibration Attention Units. The elementary gating and element-wise operations are adopted building blocks; the contribution lies in their bidirectional cross-level arrangement. Deep semantic features guide shallow-feature noise suppression, shallow spatial details calibrate deep-feature localization, and a third unit performs joint recalibration.
(4) We evaluate RADFRNet through cross-dataset comparisons, component ablations, class-wise analysis, and accuracy–efficiency measurements. On BIT-SIRST and FLIR-ADAS-v2, RADFRNet improves mAP@0.5 over YOLOv11-n by 4.4 and 8.5 percentage points, respectively. The revised analysis reports the mixed C3DEConv ablation results and treats parameter and latency increases as explicit deployment costs.

## 2. Materials and Methods

### 2.1. Related Work

#### 2.1.1. Advances in Infrared Small-Target Detection

Traditional infrared small-target methods rely on handcrafted operators for target enhancement and background separation. Morphological filtering, local contrast measures, and low-rank sparse decomposition can be interpretable and effective in constrained scenes [11,12,13,14]. Their generalization is nevertheless limited under fluctuating clouds, structured backgrounds, and strong noise.

Recent deep-learning-based infrared small-target research spans segmentation-oriented IRSTD networks, detection-oriented frameworks, scale-aware optimization, saliency modeling, representation learning, and cross-level feature fusion. DNANet, UIU-Net, MTU-Net, MRF3Net, CRPE-Net, and NS-FPN preserve weak target responses through dense interaction, nested U-shaped structures, transformer context, multi-receptive-field perception, cross-layer relative-position modeling, or frequency-domain noise suppression [15,16,17,18,19,20]. Additional recent directions include transformer-enhanced dense nested attention, multiframe spatio-temporal fusion, frequency–spatial attention, and native-resolution feature selection [21,22,23,24]. Liu et al. further introduced scale- and location-sensitive modeling for infrared small-target detection [25]. Wu et al. steered target enhancement with learnable saliency kernels [26], while self-supervised multiview infrared representation learning was explored for fine-grained object recognition [27]. Adaptive processing has also been investigated in an adjacent remote-sensing restoration task. Hou et al. proposed ACA-Net, a two-stage thick-cloud-removal framework that dynamically adjusts attention, high–low-frequency emphasis, and temporal prompting according to cloud-occlusion conditions [28]. Although ACA-Net does not perform infrared target localization, its scene-conditioned feature processing provides a relevant methodological reference for adaptive fusion under variable degradation. Together, these studies broaden the design space from local contrast enhancement to scale-aware optimization, learnable kernels, multiview representation learning, multi-receptive-field fusion, transformer-based cross-layer interaction, and condition-adaptive remote-sensing feature processing.

Detection-oriented infrared methods include IRSDT, YOLO-MST, and Infra-YOLO [9,29,30]. Lightweight YOLOv11-based multi-scale detection has also been investigated in LCW-YOLO [31]. IRSDT strengthens multi-scale representations and temporal stability, YOLO-MST combines super-resolution with multi-scale detection, and Infra-YOLO integrates attention, enhanced feature fusion, and model compression. Feature-pyramid approaches such as AFFPN and NS-FPN further improve cross-level fusion or suppress high-frequency background noise [20,32]. MPANet uses multi-patch attention, a pixel-wise attention network strengthens target-region responses, and PASSNet performs perception-aware semantic strengthening [7,33,34].

These methods cannot all be evaluated under a single controlled protocol. YOLO-MST and Infra-YOLO are the closest task matches because they produce bounding boxes. However, during the July 2026 revision audit, we could not obtain complete executable official implementations containing the model configurations, training code, and pretrained weights; we therefore did not introduce unaudited unofficial reconstructions. DNANet, UIU-Net, MTU-Net, NS-FPN, MPANet, MRF3Net, CRPE-Net, and related IRSTD methods rely primarily on pixel-level masks or segmentation-assisted supervision and are commonly evaluated with IoU, probability of detection, and false-alarm rate. In contrast, the BIT-SIRST and FLIR-ADAS-v2 experiments in this study use multi-class bounding-box annotations and report mAP and F1. Generating artificial rectangular masks from bounding boxes, or replacing the original segmentation heads and losses with a detector, would materially change those methods rather than reproduce them faithfully. ACA-Net instead restores cloud-free optical remote-sensing images and reports restoration metrics such as PSNR and SSIM. Table 1 therefore provides a task-aware comparison without conflating incompatible supervision, outputs, or metrics.

Attention-based methods collectively show the value of preserving weak-target responses in cluttered backgrounds. The Recalibration Attention Unit adopted in this work originates from the Selective Boundary Aggregation design of DuAT, where low-level boundary features and high-level semantic features are jointly recalibrated for medical-image segmentation [35]. The present contribution does not claim the elementary RAU operations as new; it lies in arranging three adopted units for bidirectional cross-level fusion at the six FPN–PAN nodes of a multi-class detector.

The review above motivates two detector-specific modifications. C3DEConv adapts an existing detail-enhanced convolution to the split–transform–concatenate structure of C3K2, while AFRE replaces direct shallow–deep concatenation with reciprocal recalibration. The distinction from prior operators and attention modules is described explicitly in Section 2.2.3 and Section 2.2.4.

#### 2.1.2. Evolution of YOLO-Based Detection Algorithms

The YOLO family is widely used for real-time object detection. YOLO-based infrared detectors have introduced super-resolution, multi-scale observation, attention, and feature-pyramid modifications. YOLO-MST integrates super-resolution and multi-scale observation, whereas Infra-YOLO combines attention, enhanced feature fusion, and model compression [29,30].

Table 2 summarizes the architectural differences among the representative YOLO baselines used to motivate the selection of YOLOv11-n.

Compared with earlier YOLO variants, YOLOv11-n uses C3K2-based aggregation and C2PSA within an anchor-free detection framework. Nevertheless, C3K2 is not explicitly designed to preserve the weak gradient cues of extremely small infrared targets, and direct cross-level fusion in the original neck may propagate shallow background clutter. We therefore modify the backbone and neck and extend the prediction topology with a high-resolution P2 branch, while retaining the decoupled-head formulation used by YOLOv11-n.

### 2.2. Proposed Method

#### 2.2.1. Design Motivation

RADFRNet is designed around two complementary failure modes of YOLOv11-n in infrared small-target detection. The first is progressive detail loss in the backbone: weak target boundaries may be attenuated after repeated convolution and downsampling. The second is semantic-spatial mismatch in the neck: shallow features retain boundary cues but also contain clutter, whereas deep features provide stronger semantics but coarser localization. C3DEConv addresses the first failure mode through stage-specific edge-sensitive extraction, while AFRE addresses the second through bidirectional shallow–deep recalibration.

The correspondence between the observed failure modes and the proposed design responses is summarized in Table 3.

#### 2.2.2. Overall Architecture of RADFRNet

**Figure 1:**
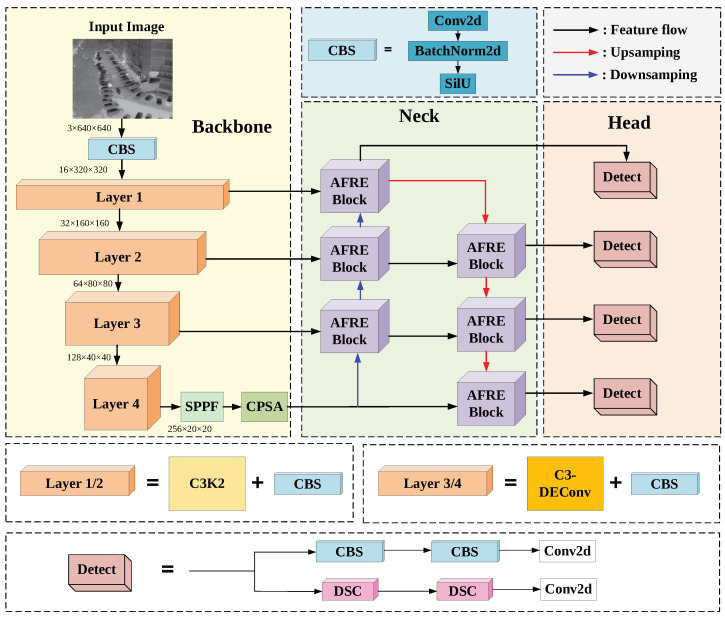
Overall architecture of RADFRNet. The backbone retains the original C3K2 modules in the early stages and replaces selected later-stage modules with C3DEConv. The neck uses six AFRE blocks at the top-down and bottom-up cross-level fusion nodes, and the prediction topology covers the P2–P5 feature levels through four decoupled detection heads. Black, red, and blue arrows denote feature flow, upsampling, and downsampling, respectively.

The overall architecture is shown in Figure 1. In the backbone, the DEConv operator originally introduced in DEA-Net [42] is embedded into selected C3K2 stages to form C3DEConv. The early C3K2 stages are retained to avoid excessive amplification of low-level background gradients. In the neck, six AFRE blocks replace direct cross-level fusion operations along the FPN–PAN paths. Each AFRE block performs bidirectional interaction between aligned shallow and deep features before joint recalibration. In addition to the conventional P3–P5 prediction levels, the native topology introduces a high-resolution P2 branch, yielding four-scale P2–P5 detection. The prediction heads retain the decoupled YOLOv11-n formulation, but the detection topology is therefore extended rather than left unchanged.

The input image first passes through the original YOLOv11-n convolution–batch-normalization–SiLU stem. The backbone contains four feature-extraction stages. Stages 1 and 2 retain C3K2, whereas Stages 3 and 4 use C3DEConv. This is the submitted detector configuration: the early stages retain the original aggregation to avoid indiscriminate amplification of low-level gradients, while the later selected stages introduce edge-sensitive extraction after preliminary semantic features have formed. We do not claim that this placement is a universally optimal stage configuration.

#### 2.2.3. C3DEConv Backbone

**Figure 2:**
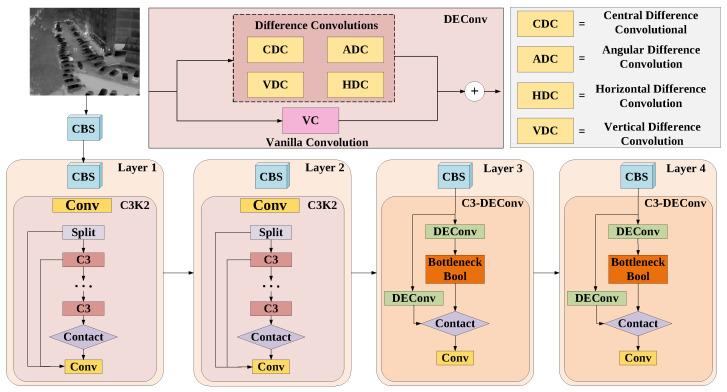
Integration of the adopted DEConv operator into the C3K2 aggregation structure. The training-time DEConv operator contains standard, central-difference, angular-difference, horizontal-difference, and vertical-difference convolution branches. In C3DEConv, the operator replaces selected convolutions inside the C3K2-compatible block at Stages 3 and 4. Solid arrows indicate feature flow, the dashed rectangle groups the four difference-convolution branches, and the ellipsis denotes repeated C3 units omitted for compactness. Colors distinguish operation types only and do not encode quantitative values.

Figure 2 shows how the adopted DEConv operator is integrated into the selected C3K2 stages. YOLOv11-n uses C3K2 as a compact CSP-style aggregation module. Although C3K2 provides efficient feature extraction, its standard convolutions are not explicitly optimized for the weak gradient and boundary cues of tiny infrared targets. DEConv was originally proposed in DEA-Net for image dehazing and detail enhancement [42]. We adopt the DEConv formulation without claiming the operator itself as a new contribution. In this work, DEConv is embedded into the split–transform–concatenate structure of C3K2 to form C3DEConv. The distinction from the original DEA-Net use is threefold: (1) DEConv operates inside a CSP-style detection block rather than as an independent image-restoration layer; (2) it is deployed only at selected backbone stages; and (3) it is optimized jointly for class prediction and bounding-box regression rather than pixel reconstruction.

For an input feature map *X*, the equivalent deployed convolution is written in Equation (1) as

(1) $$Y=X*K_{\mathrm{eq}}+b_{\mathrm{eq}},$$

where ∗ denotes convolution. The equivalent kernel in Equation (2) is obtained by transforming and summing the training-time branch kernels:

(2) $$K_{\mathrm{eq}}=K_{\mathrm{VC}}+T_{\mathrm{CDC}}\left(K_{\mathrm{CDC}}\right)+T_{\mathrm{ADC}}\left(K_{\mathrm{ADC}}\right)+T_{\mathrm{HDC}}\left(K_{\mathrm{HDC}}\right)+T_{\mathrm{VDC}}\left(K_{\mathrm{VDC}}\right).$$

Because the branch operations are linear and share compatible strides, padding, grouping, and output dimensions, their transformed kernels can be summed into one equivalent kernel for deployment. The deployed layer does not execute the five branches separately. This removes branch-wise execution overhead relative to the equivalent deployed convolution; it does not imply that the complete C3DEConv-based detector has the same parameter count, FLOPs, or latency as the original C3K2-based YOLOv11-n model.

Table 4 states the innovation boundary between the original operator and its detector-specific integration.

#### 2.2.4. AFRE Fusion Design

**Figure 3:**
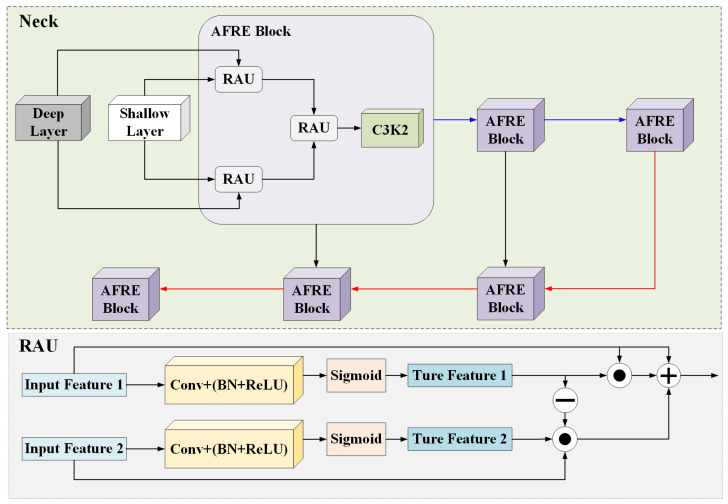
Adaptive Feature Recalibration (AFRE) block. Two RAUs perform reciprocal deep-to-shallow and shallow-to-deep recalibration, and a third RAU jointly refines the two outputs. The minus sign denotes reverse weighting, the plus sign denotes element-wise addition, and the filled dot denotes element-wise multiplication.

The AFRE topology and the adopted RAU operations are illustrated in Figure 3. Direct concatenation in the original FPN–PAN neck treats shallow and deep features as equally useful, although they contain different information. Shallow features retain target boundaries but also include background texture, whereas deep features contain stronger semantics but may exhibit spatial imprecision for extremely small targets. AFRE is introduced as a cross-level fusion block to address this mismatch rather than as another single-feature attention module.

AFRE is constructed from three Recalibration Attention Units (RAUs) adopted from the Selective Boundary Aggregation module of DuAT [35]. The elementary operations used by RAU—convolutional projection, sigmoid gating, element-wise multiplication, and reverse weighting—are not individually claimed as new. The contribution of AFRE lies in its detector-specific three-unit bidirectional arrangement: one branch uses deep semantics to suppress irrelevant shallow responses, the second uses shallow spatial details to calibrate deep responses, and the third performs joint recalibration at each FPN–PAN fusion node.

Six AFRE blocks are used because the YOLOv11-n neck contains three top-down and three bottom-up cross-level fusion nodes. At each node, the two inputs are aligned by the resolution-conversion and channel-projection operations associated with that neck node, and AFRE replaces direct concatenation. This placement ties the number of AFRE blocks to the neck topology rather than treating it as an independent hyperparameter.

Within each RAU, the two aligned input features are projected through convolution, batch normalization, and ReLU, followed by sigmoid gating. The gated responses are combined using element-wise multiplication, reverse weighting, and addition, as illustrated in Figure 3. The first two RAUs implement deep-to-shallow and shallow-to-deep guidance, and the third RAU jointly recalibrates the two refined responses. This description follows the computation graph used in the submitted implementation and avoids presenting the adopted elementary operations as a new attention formulation.

ECA recalibrates channels within one feature map, CBAM sequentially applies channel and spatial weighting to one feature map, and Non-local attention models long-range self-interaction. AFRE instead receives two cross-level feature maps and performs reciprocal guidance before joint fusion. Table 5 summarizes the design differences and reports a controlled computational-cost comparison under a common implementation and timing protocol.

Under this controlled protocol, ECA adds almost no parameters, CBAM introduces a moderate parameter increase, and Non-local attention has the largest FLOP increase. AFREAdapter has the largest parameter and latency increments among the tested fusion alternatives, while its FLOP increment remains substantially below that of Non-local attention. The result therefore supports an explicit accuracy–efficiency trade-off rather than a negligible-overhead claim.

## 3. Experiments and Results

### 3.1. Datasets

BIT-SIRST is a public infrared dataset that was extended in this study to four-class bounding-box detection with car, person, UAV, and ship categories. The experimental split retained for this study contains 10,478 images: 7334 for training, 2096 for validation, and 1048 for testing. These counts are taken directly from the archived split lists and replace the inconsistent total reported in an earlier draft.

Two annotators with infrared-image interpretation experience participated in manual label preparation and cross-checking. The retained annotation archive does not preserve independently paired annotation files, a predefined box-matching IoU threshold, or per-case disagreement and adjudication logs. Consequently, the earlier statement of “over 95% consistency” has been removed, and no numerical inter-annotator agreement is claimed in the revised manuscript. The final four-class labels used for training and evaluation are treated as the consensus annotation set. This conservative description avoids reporting an agreement statistic that cannot be reproduced from the available records.

FLIR-ADAS-v2 is a public thermal-infrared driving dataset containing 14,452 thermal images [43]. The experiments use a study-specific subset of 10,478 images, comprising 7334 training, 1048 validation, and 2096 test images. The formal eight-class label set is car, truck, bus, person, bike, light, other vehicle, and motor; sign and hydrant annotations are excluded while the archived image split is preserved. Although the BIT-SIRST and FLIR experimental sets contain the same number of images, they are independent datasets from different sources and share no imagery; the identical totals arise from the study-specific split files retained for the two experiments. FLIR-ADAS-v2 provides a ground-driving setting with occlusion, clutter, and broader target-scale variation. Results on the two datasets are therefore treated as evidence for two distinct settings rather than proof of universal cross-domain robustness. For the additional diagnostic analysis, the baseline and C3DEConv-only variants were retrained under the same audited eight-class protocol, with identical data splits, initialization, image size, batch size, optimizer, training length, random seed, and test procedure. These results are reported separately from the original four-model ablation series.

### 3.2. Evaluation Metrics

A prediction is counted as a true positive when it matches a ground-truth box at an IoU threshold of 0.5 for the reported precision, recall, and F1 score. Precision and recall are defined as

(3) $$P=\frac{TP}{TP+FP},\;\;R=\frac{TP}{TP+FN},$$

and the F1 score is

(4) $$F_{1}=2\frac{PR}{P+R}.$$

For class *c*, average precision is the area under the precision–recall curve. Mean average precision is defined as

(5) $$\mathrm{mAP}=\frac{1}{N_{c}}\sum_{c=1}^{N_{c}}{\mathrm{AP}}_{c},$$

where $N_{c}$ is the number of classes. We report mAP@0.5 and mAP@0.5:0.95 using the same evaluation script for all compared models. For the controlled FLIR re-evaluation, F1 is summarized in two complementary ways: the macro-average of the eight class-wise F1 scores at a fixed confidence threshold of 0.25, and the model-specific optimum obtained from the F1–confidence curve. Reporting both values separates operating-point sensitivity from threshold-independent AP behavior.

### 3.3. Experimental Settings and Hyperparameters

All models are implemented in PyTorch 2.5.1 with CUDA 12.1 and trained on an NVIDIA GeForce RTX 4060 GPU (NVIDIA Corporation, Santa Clara, CA, USA) under Windows 11. Shared training settings are summarized in Table 6: 100 epochs, batch size 8, input resolution 640 × 640, SGD with an initial learning rate of 0.01, momentum 0.9, weight decay 0.0005, four workers, and Mosaic and Mixup augmentation. The ablation variants use the same data split and training schedule.

All latency entries below are per-image GPU inference times obtained under the original 640 × 640 setup used for the corresponding dataset. Nominal throughput is calculated as 1000/latency, and latency comparisons are made within each dataset under the same inference configuration. Because BIT-SIRST and FLIR-ADAS-v2 use different class configurations in the detection head, the reported model-complexity values differ slightly between datasets.

### 3.4. Comparative Experiments

The controlled quantitative tables include only models implemented and evaluated under the same multi-class bounding-box protocol. YOLO-MST and Infra-YOLO are task-compatible in principle, but complete executable official implementations were not obtained during the revision audit. To avoid unverifiable unofficial reconstructions, they are discussed in the task-aware comparison rather than assigned numerical results. DNANet, UIU-Net, MTU-Net, NS-FPN, and MPANet require pixel-level masks or segmentation-assisted supervision, whereas the present BIT-SIRST and FLIR-ADAS-v2 annotations are multi-class bounding boxes. Their published IoU, probability-of-detection, and false-alarm values are therefore not inserted into Table 7 and Table 8; generating artificial masks or replacing their original heads would change the evaluated methods. ACA-Net is likewise retained as an adjacent remote-sensing restoration reference rather than a detector baseline. Direct performance claims are restricted to models evaluated under the common bounding-box protocol in this study. RT-DETR, RepViT, and CSWin-Transformer are included as representative real-time transformer and efficient-backbone baselines [44,45,46].

#### 3.4.1. BIT-SIRST

**Figure 4:**
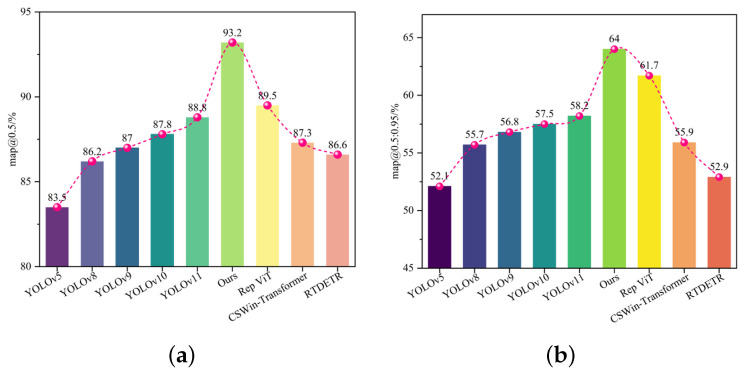

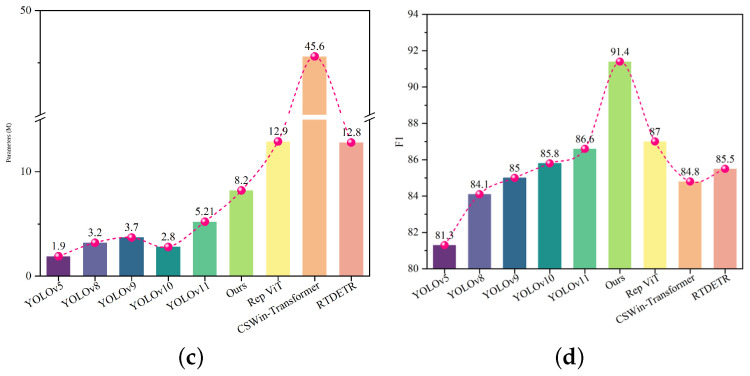
Metric comparison on BIT-SIRST: (**a**) mAP@0.5; (**b**) mAP@0.5:0.95; (**c**) parameters; and (**d**) F1 score.

Table 7 and Figure 4 show that RADFRNet improves mAP@0.5 by 4.4 percentage points over YOLOv11-n. Under the same original GPU inference setup, latency increases from 3.3 to 6.7 ms, an absolute increase of 3.4 ms and a 2.03× ratio. The corresponding nominal throughput changes from approximately 303 to 149 FPS, while the reported model complexity rises from 5.21 M to 8.2 M. RADFRNet therefore preserves high-rate GPU inference, but the accuracy gain is accompanied by a clear latency and complexity cost.

#### 3.4.2. FLIR-ADAS-v2

**Figure 5:**
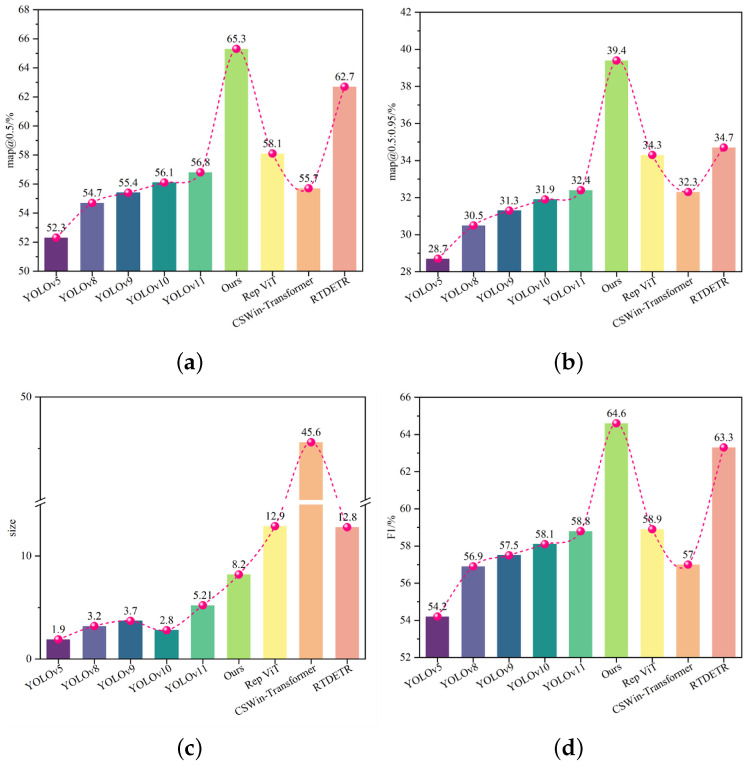
Metric comparison on FLIR-ADAS-v2: (**a**) mAP@0.5; (**b**) mAP@0.5:0.95; (**c**) parameters; and (**d**) F1 score.

Table 8 and Figure 5 show that RADFRNet improves mAP@0.5 by 8.5 percentage points over YOLOv11-n and by 2.6 points over RT-DETR. Under the same original GPU inference setup, latency increases from 3.0 to 7.8 ms, corresponding to a 2.60× ratio and a nominal throughput change from approximately 333 to 128 FPS. The reported model complexity increases from 5.5 M to 8.6 M. The model therefore retains high-rate GPU inference, although the additional cost remains material for embedded or power-constrained deployment.

#### 3.4.3. Qualitative Results

**Figure 6:**
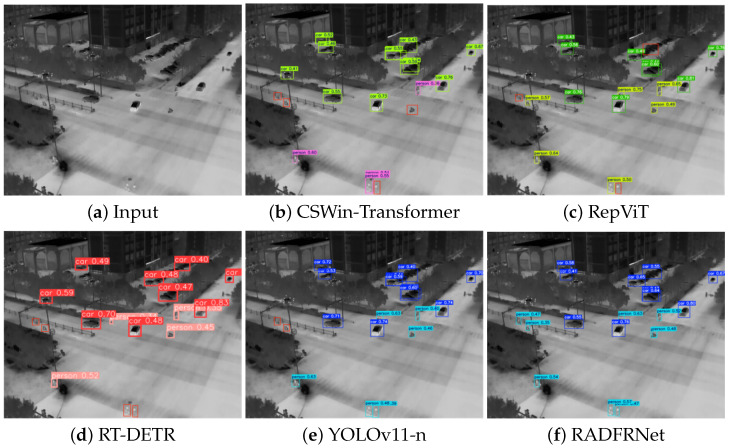
Visual comparison in Scene 1. The qualitative figure is interpreted as illustrative evidence rather than a substitute for class-wise false-positive and false-negative statistics.

**Figure 7:**
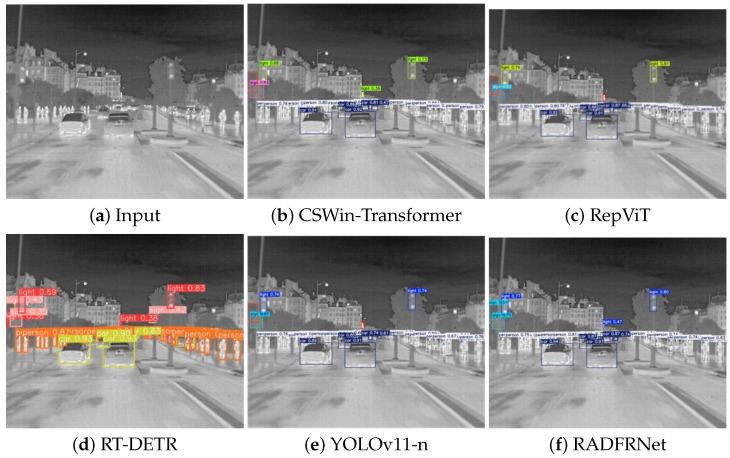
Visual comparison in Scene 2.

**Figure 8:**
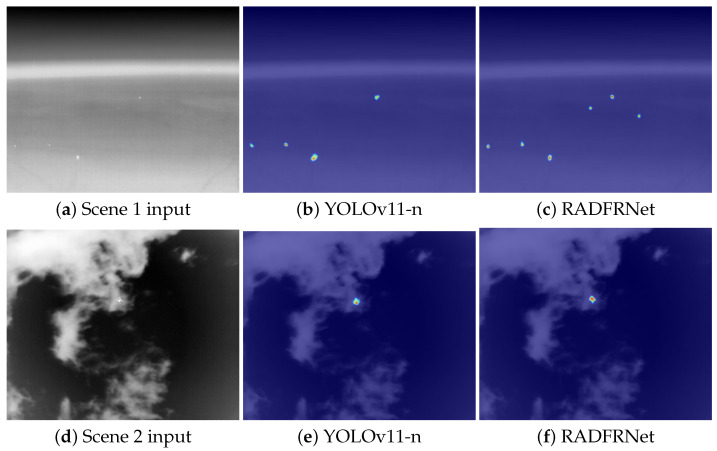
Heatmap comparison. Warmer colors indicate higher activation.

Figure 6 and Figure 7 provide qualitative comparisons in two representative scenes, while Figure 8 compares baseline and RADFRNet activation maps. These examples are illustrative and are not used as substitutes for quantitative false-positive and false-negative statistics.

### 3.5. Ablation Study

#### 3.5.1. C3DEConv Module

Table 9 summarizes the class-dependent changes introduced by C3DEConv. C3DEConv increases aggregate mAP@0.5 from 0.888 to 0.896 and mAP@0.5:0.95 from 0.583 to 0.594, but the changes are not uniform. For person and ship, F1 decreases by 0.020 even though both AP measures improve, indicating that performance at the selected confidence operating point deteriorates while ranking across the full precision–recall curve improves. For UAV, F1 increases by 0.042, whereas mAP@0.5 and mAP@0.5:0.95 decrease slightly by 0.006 and 0.005. These mixed changes show that C3DEConv alters classification confidence and localization behavior rather than uniformly improving every class and metric. The available aggregate and class-wise results do not support the earlier claim that the module always suppresses background interference.

#### 3.5.2. AFRE Ablation

Table 10 shows that AFRE provides a larger and more consistent aggregate gain than C3DEConv alone, increasing F1 by 0.044, mAP@0.5 by 0.037, and mAP@0.5:0.95 by 0.056. This ablation establishes effectiveness relative to direct YOLOv11-n fusion but does not, by itself, establish superiority over ECA, CBAM, or Non-local attention; the controlled design and cost comparison is therefore reported separately in Table 5.

#### 3.5.3. Combined Ablations

**Figure 9:**
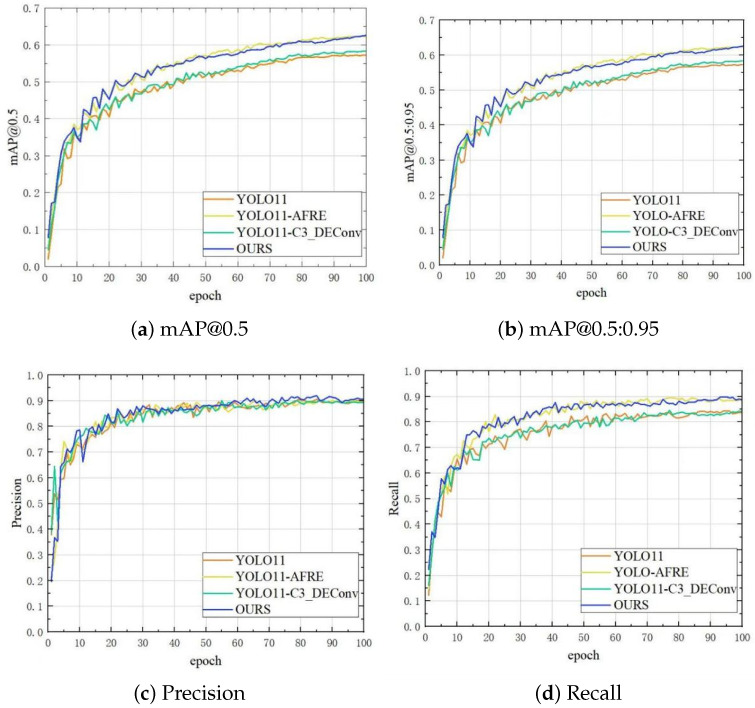
Comparison of ablated structures on BIT-SIRST.

**Figure 10:**
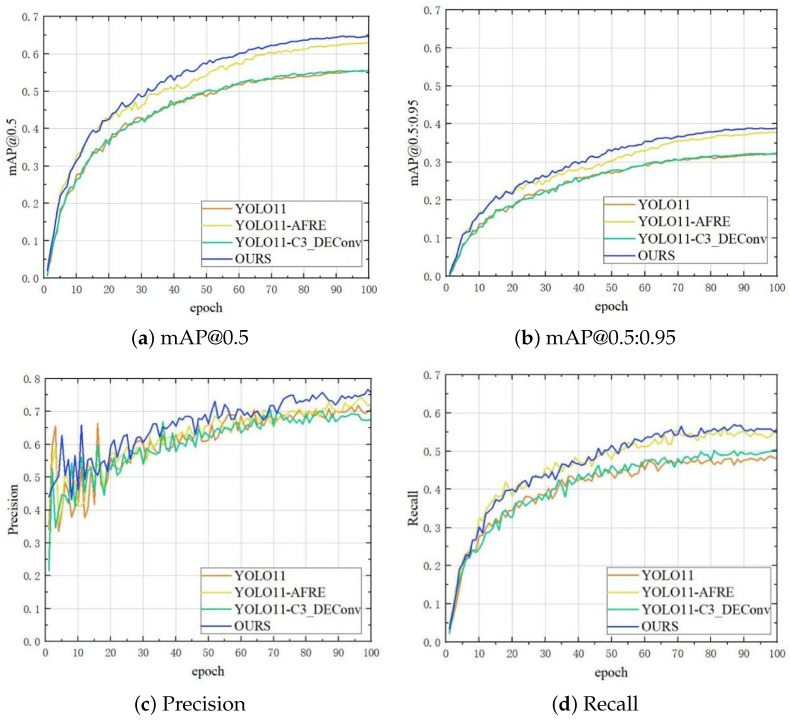
Comparison of ablated structures on FLIR-ADAS-v2.

Table 11 and Table 12, together with Figure 9 and Figure 10, summarize the combined ablations. On FLIR-ADAS-v2, C3DEConv alone decreases F1 from 0.588 to 0.582 and mAP@0.5 from 0.568 to 0.566, while mAP@0.5:0.95 increases from 0.324 to 0.327. This is a negative/mixed result and is reported explicitly rather than attributed to background suppression without evidence. AFRE provides the dominant aggregate improvement, and the combined model achieves the highest aggregate values at the cost of higher parameter count and latency.

The controlled re-evaluation clarifies the role of the confidence operating point. At the model-specific optimum, F1 increases from 0.6698 to 0.6786. At the common threshold of 0.25, however, macro-F1 decreases from 0.6604 to 0.6553 (−0.0052), closely matching the direction and magnitude of the −0.006 change in the original ablation. The AP measures also decrease slightly, by 0.0017 at IoU 0.5 and by 0.0039 across IoU 0.5:0.95. Table 13 summarizes the aggregate controlled results, and Table 14 shows that the fixed-threshold decline is concentrated in several categories. Bus exhibits the largest reduction (ΔF1=−0.0247, $\Delta {\mathrm{AP}}_{50}=-0.0142$), with 10 additional false positives and 11 additional false negatives. Bike, motor, truck, and person also show lower fixed-threshold F1, while car, light, and other vehicle improve slightly. Person has 59 additional false negatives but 16 fewer false positives, indicating a recall-oriented shift rather than a general increase in spurious detections. The contrast between fixed-threshold and optimal-threshold F1 indicates threshold-sensitive confidence calibration. It does not establish a causal background-edge mechanism. Because this controlled re-evaluation is an independent experimental series, its absolute values are reported separately and are not merged with the original four-model ablation table.

## 4. Discussion

The two modules play different roles. C3DEConv provides modest aggregate gains on BIT-SIRST but has class-dependent effects, and it does not improve every aggregate metric on FLIR-ADAS-v2 when used alone. The BIT-SIRST class-wise results show threshold-dependent trade-offs: person and ship obtain lower F1 despite higher AP, while UAV obtains higher F1 but slightly lower AP. AFRE contributes the larger and more consistent aggregate gain by recalibrating shallow and deep features at the neck fusion nodes. The combined model achieves the highest aggregate accuracy in the reported ablations, at the cost of increased parameters and latency.

The qualitative detection maps and heatmaps illustrate representative behavior but do not independently prove lower missed-detection rates or universal robustness. Table 13 and Table 14 provide the corresponding controlled eight-class evidence. The model-specific optimal F1 improves, whereas fixed-threshold macro-F1 and both AP metrics decrease slightly. The largest fixed-threshold degradation occurs for bus, followed by bike and motor; person shows a smaller F1 reduction associated mainly with increased false negatives. These results indicate that C3DEConv changes confidence calibration and class-wise operating behavior rather than producing a uniform gain or loss. No causal background-edge mechanism is inferred.

### Limitations and Future Work

The present study has two deployment-related limitations. First, the measured GPU latency ratios are 2.03× and 2.60× relative to YOLOv11-n on BIT-SIRST and FLIR-ADAS-v2, respectively. Although the corresponding nominal throughputs of 149 and 128 FPS remain suitable for high-rate GPU inference, edge-device latency and energy consumption have not yet been evaluated. Second, evaluation on two datasets does not establish robustness to sensor changes, atmospheric conditions, weather, or unseen domains. The model also processes single frames and does not use temporal continuity. Future work will investigate lightweight cross-level recalibration, edge-device deployment, infrared video modeling, visible–infrared fusion, and domain-shift evaluation.

## 5. Conclusions

This study presents RADFRNet for multi-class infrared small-target detection. The model embeds the previously proposed DEConv operator into selected C3K2 stages to form a detection-oriented C3DEConv backbone. The contribution of this component lies in structural and stage-specific adaptation, not in a new differential-convolution formulation. C3DEConv enhances edge-sensitive feature extraction, although its independent effect is class- and dataset-dependent. The AFRE block adapts three RAUs into a bidirectional cross-level detector module and provides the larger aggregate improvement in the reported ablations.

On BIT-SIRST and FLIR-ADAS-v2, RADFRNet improves mAP@0.5 over YOLOv11-n by 4.4 and 8.5 percentage points, respectively. These gains are accompanied by increased model complexity and higher latency. The reported model-complexity values are 8.2 M and 8.6 M, and the per-image latencies are 6.7 and 7.8 ms, corresponding to nominal throughputs of approximately 149 and 128 FPS under the original 640×640 GPU inference setup. RADFRNet therefore maintains high-rate GPU inference, but it is an accuracy-oriented detector rather than a latency-neutral or strictly lightweight replacement for YOLOv11-n.

The evaluation is limited to two infrared datasets and GPU-based single-frame inference. Future work will focus on lightweight recalibration, edge-device deployment, temporal information, multimodal fusion, and robustness under sensor noise and atmospheric disturbance. 

## Figures and Tables

**Table 1:** Task-aware comparison of representative infrared small-target and adjacent remote-sensing methods.

Method	Primary Output	Relation to the Present Study
YOLO-MST/Infra-YOLO	Bounding boxes	Closest task match; complete executable official implementations were not obtained during revision, so no unaudited reimplementation was used.
DNANet/UIU-Net/MTU-Net	Pixel masks	Require pixel-level mask supervision; the present datasets use multi-class boxes, and box-to-mask conversion or head replacement would alter the original protocols.
NS-FPN/MPANet	Pixel masks	Segmentation-oriented or mask-supervised IRSTD methods; their mask labels and IoU/$P_{d}$/$F_{a}$ metrics are not interchangeable with bounding-box mAP/F1.
Scale- and location-sensitive IRSTD	Pixel masks	Introduces scale- and location-aware optimization for tiny targets under a segmentation protocol.
Saliency with learnable kernels	Pixel masks	Uses learnable local-saliency modeling to distinguish weak targets from structured backgrounds.
MRF3Net	Pixel masks	Combines multi-receptive-field perception with effective feature fusion.
CRPE-Net	Pixel masks	Uses cross-layer relative-position embedding in a transformer-based detector.
Multiview self-supervised infrared recognition	Image-level classes	Provides relevant infrared representation-learning evidence but does not perform target localization.
ACA-Net	Cloud-free RS images	Adjacent remote-sensing restoration method; adaptive attention, frequency processing, and temporal prompting are relevant to dynamic fusion, but PSNR/SSIM are not comparable with detection mAP.
RADFRNet	Multi-class boxes	Integrates C3DEConv and bidirectional AFRE fusion under the bounding-box protocol used in this study.

**Table 2:** Core differences among representative YOLO versions.

Version	Backbone Core	Neck	Head	Key Characteristic
YOLOv5 [36]	CSPDarknet with C3	FPN + PAN	Anchor-based coupled head	Mature engineering baseline.
YOLOv8 [37]	C2f-based CSP structure	FPN + PAN with C2f	Anchor-free decoupled head	Efficient anchor-free baseline.
YOLOv9 [38]	GELAN with PGI	FPN + PAN	Decoupled head with auxiliary supervision	Gradient-routing design.
YOLOv10 [39]	Lightweight CSP design	Lightweight FPN + PAN	End-to-end NMS-free head	Low-latency end-to-end detection.
YOLOv11-n [40,41]	C3K2 and C2PSA	FPN + PAN	Anchor-free decoupled head	Baseline selected for the present study.

**Table 3:** Problem-to-design mapping in RADFRNet.

Observed Failure Mode	Corresponding Design Response
Weak target texture and boundary attenuation	Stage-specific C3DEConv in the backbone.
Shallow background clutter	Deep-to-shallow semantic guidance in AFRE.
Deep-feature localization shift	Shallow-to-deep spatial calibration in AFRE.
Unequal cross-level contribution	Third-RAU joint recalibration before output fusion.

**Table 4:** Distinction between the original DEA-Net DEConv operator and C3DEConv in this work.

Aspect	DEA-Net DEConv	C3DEConv in This Work
Original task	Image dehazing/detail restoration	Multi-class infrared bounding-box detection
Basic operator	Original DEConv formulation	Same adopted DEConv formulation
Structural context	Restoration-network convolution layer	Embedded inside C3K2/CSP aggregation
Deployment position	Restoration layers	Selected YOLOv11-n backbone stages
Optimization objective	Pixel reconstruction	Classification and box regression
Claimed contribution	Original operator	Structural and stage-specific adaptation

**Table 5:** Controlled computational-cost comparison of cross-level fusion mechanisms. The parameter and FLOP increments are measured relative to direct concatenation.

Module	Input Relation	Primary Mechanism	ΔParams	ΔFLOPs	FP32 Latency
Direct concat.	Two cross-level maps	Channel concatenation without explicit recalibration	0	0	10.514±1.063 ms
ECA	One feature map	Lightweight channel-only recalibration	+0.00002 M	+0.0027 G	11.206±1.385 ms
CBAM	One feature map	Sequential channel and spatial weighting	+0.39892 M	+0.0109 G	11.529±1.130 ms
Non-local ^†^	One feature map	Long-range self-interaction	+0.79462 M	+6.0714 G	12.005±0.973 ms
AFREAdapter (SBA) ^‡^	Two cross-level maps	Reciprocal shallow–deep guidance and joint recalibration	+3.97798 M	+0.0244 G	13.629±1.156 ms

The benchmark used an NVIDIA GeForce RTX 4060 GPU (NVIDIA Corporation, Santa Clara, CA, USA), batch size 1, a 640×640 input, 100 warm-up iterations, and 500 CUDA-event-timed forward passes. Image loading, preprocessing, host-to-device transfer, and NMS were excluded. ^†^ The Non-local implementation used 2× pooled key/value features. ^‡^ AFREAdapter invokes the same SBA implementation as the project AFRE module in a shape-preserving controlled wrapper; it is not numerically interchangeable with the final native six-SBA P2–P5 topology.

**Table 6:** Workstation and shared training settings.

Parameter	Value	Parameter	Value
GPU	NVIDIA GeForce RTX 4060	Operating system	Windows 11
Epochs	100	Batch size	8
Input size	640×640	Optimizer	SGD
Initial learning rate	0.01	Momentum	0.9
Weight decay	0.0005	Workers	4
Augmentation	Mosaic and Mixup	Model scale	Nano (n)

**Table 7:** Comparative results on BIT-SIRST.

Method	Backbone	mAP@0.5	mAP@0.5:0.95	Latency	Params	F1
YOLOv5	CSPDarknet53	83.5%	52.1%	2.1 ms	1.9 M	81.3%
YOLOv8	CSPDarknet + C2f	86.2%	55.7%	2.7 ms	3.2 M	84.1%
YOLOv9	GELAN	87.0%	56.8%	3.0 ms	3.7 M	85.0%
YOLOv10	Lightweight CSP	87.8%	57.5%	2.4 ms	2.8 M	85.8%
YOLOv11-n	C3K2 baseline	88.8%	58.3%	3.3 ms	5.21 M	86.6%
RT-DETR	EfficientViT	86.6%	52.9%	8.0 ms	12.8 M	85.5%
RepViT	RepViT-M0.9	89.5%	61.7%	7.5 ms	12.9 M	87.0%
CSWin-Transformer	CSWin-Tiny	87.3%	55.9%	7.7 ms	45.6 M	84.8%
RADFRNet	C3DEConv + AFRE	**93.2%**	**64.0%**	6.7 ms	8.2 M	**91.4%**

Note: Bold values indicate the highest accuracy in the corresponding metric column.

**Table 8:** Comparative results on FLIR-ADAS-v2.

Method	Backbone	mAP@0.5	mAP@0.5:0.95	Latency	Params	F1
YOLOv5	CSPDarknet53	52.3%	28.7%	1.9 ms	1.9 M	54.2%
YOLOv8	CSPDarknet + C2f	54.7%	30.5%	2.5 ms	3.2 M	56.9%
YOLOv9	GELAN	55.4%	31.3%	2.8 ms	3.7 M	57.5%
YOLOv10	Lightweight CSP	56.1%	31.9%	2.3 ms	2.8 M	58.1%
YOLOv11-n	C3K2 baseline	56.8%	32.4%	3.0 ms	5.5 M	58.8%
RT-DETR	EfficientViT	62.7%	34.7%	8.5 ms	12.8 M	63.3%
RepViT	RepViT-M0.9	58.1%	34.3%	6.0 ms	12.9 M	58.9%
CSWin-Transformer	CSWin-Tiny	55.7%	32.3%	8.3 ms	45.6 M	57.0%
RADFRNet	C3DEConv + AFRE	**65.3%**	**39.4%**	7.8 ms	8.6 M	**64.6%**

Note: Bold values indicate the highest accuracy in the corresponding metric column.

**Table 9:** Class-wise C3DEConv ablation on BIT-SIRST.

C3DEConv	Class	Instances	F1	mAP@0.5	mAP@0.5:0.95
×	All	4332	0.866	0.888	0.583
×	Car	1181	0.788	0.816	0.479
×	Person	180	0.840	0.840	0.477
×	UAV	1412	0.901	0.956	0.769
×	Ship	1559	0.917	0.942	0.605
✓	All	4332	0.867	0.896	0.594
✓	Car	1181	0.808	0.833	0.493
✓	Person	180	0.820	0.855	0.510
✓	UAV	1412	0.943	0.950	0.764
✓	Ship	1559	0.897	0.947	0.610

Note: ✓ and × indicate that the corresponding component is enabled and disabled, respectively.

**Table 10:** Class-wise AFRE ablation on BIT-SIRST.

AFRE	Class	Instances	F1	mAP@0.5	mAP@0.5:0.95
×	All	4332	0.866	0.888	0.583
×	Car	1181	0.788	0.816	0.479
×	Person	180	0.840	0.841	0.477
×	UAV	1412	0.901	0.956	0.769
×	Ship	1559	0.917	0.942	0.605
✓	All	4332	0.910	0.925	0.639
✓	Car	1181	0.856	0.894	0.541
✓	Person	180	0.879	0.876	0.525
✓	UAV	1412	0.972	0.963	0.844
✓	Ship	1559	0.933	0.966	0.646

Note: ✓ and × indicate that the corresponding component is enabled and disabled, respectively.

**Table 11:** Ablation study on BIT-SIRST.

YOLOv11-n	C3DEConv	AFRE	F1	${\mathrm{mAP}}_{50}$	${\mathrm{mAP}}_{50:95}$	Params
✓	×	×	0.866	0.888	0.583	5.21 M
✓	✓	×	0.867	0.896	0.594	5.70 M
✓	×	✓	0.910	0.925	0.639	7.72 M
✓	✓	✓	0.914	0.932	0.640	8.20 M

Note: ✓ and × indicate that the corresponding component is enabled and disabled, respectively.

**Table 12:** Ablation study on FLIR-ADAS-v2.

YOLOv11-n	C3DEConv	AFRE	F1	${\mathrm{mAP}}_{50}$	${\mathrm{mAP}}_{50:95}$	Params
✓	×	×	0.588	0.568	0.324	5.50 M
✓	✓	×	0.582	0.566	0.327	6.00 M
✓	×	✓	0.644	0.645	0.388	8.10 M
✓	✓	✓	0.646	0.653	0.394	8.60 M

Note: ✓ and × indicate that the corresponding component is enabled and disabled, respectively.

**Table 13:** Controlled eight-class FLIR re-evaluation of the baseline and C3DEConv-only models. $F1_{\mathrm{opt}}$ is obtained at each model’s optimal F1 operating point, whereas $F1_{0.25}$ is the macro-average of the eight class-wise F1 scores at a fixed confidence threshold of 0.25.

Model	$F1_{\mathrm{opt}}$	$F1_{0.25}$	${\mathrm{mAP}}_{50}$	${\mathrm{mAP}}_{50:95}$	FLOPs
YOLOv11-n baseline	0.6698	0.6604	0.6835	0.4231	6.3205 G
C3DEConv-only	0.6786	0.6553	0.6817	0.4192	6.3218 G
Change	+0.0087	−0.0052	−0.0017	−0.0039	+0.0013 G

**Table 14:** Class-wise quantitative breakdown from the controlled eight-class FLIR re-evaluation at a fixed confidence threshold of 0.25. Δ denotes C3DEConv-only minus the corresponding baseline result; AP and error-count changes are computed from the same test set.

Class	Baseline F1	C3DEConv F1	ΔF1	$\Delta {\mathrm{AP}}_{50}$	ΔFP	ΔFN
Car	0.7599	0.7609	+0.0009	+0.0004	−65	+18
Truck	0.5886	0.5831	−0.0055	−0.0015	−4	+3
Bus	0.7642	0.7395	−0.0247	−0.0142	+10	+11
Person	0.7304	0.7272	−0.0031	−0.0036	−16	+59
Bike	0.6583	0.6444	−0.0139	−0.0019	+33	+13
Light	0.6178	0.6215	+0.0037	−0.0062	−59	+12
Other vehicle	0.5293	0.5385	+0.0092	−0.0034	−3	−2
Motor	0.6349	0.6272	−0.0078	+0.0165	+15	−4

## Data Availability

The original BIT-SIRST and FLIR-ADAS-v2 images are available from their respective providers. The four-class BIT-SIRST bounding-box annotations, the eight-class FLIR label-remapping files, and the train/validation/test split lists used in this study are available from the corresponding author upon reasonable request.
